# Single nucleotide polymorphisms (SNPs) are highly conserved in rhesus (*Macaca mulatta*) and cynomolgus (*Macaca fascicularis*) macaques

**DOI:** 10.1186/1471-2164-8-480

**Published:** 2007-12-31

**Authors:** Summer L Street, Randall C Kyes, Richard Grant, Betsy Ferguson

**Affiliations:** 1 Genetics Research and Informatics Program, Oregon National Primate Research Center, Oregon Health & Sciences University, Beaverton, OR 97006, USA; 2 Washington National Primate Research Center, University of Washington, Seattle, WA 98195, USA; 3 Department of Psychology, University of Washington, Seattle, WA 98195, USA; 4 SNBL USA, Ltd., Everett, WA 98203, USA

## Abstract

**Background:**

*Macaca fascicularis *(cynomolgus or longtail macaques) is the most commonly used non-human  primate in biomedical research. Little is known about the genomic variation in cynomolgus macaques or how the sequence variants compare to those of the well-studied related species, *Macaca mulatta *(rhesus macaque). Previously we identified single nucleotide polymorphisms (SNPs) in portions of 94 rhesus macaque genes and reported that Indian and Chinese rhesus had largely different SNPs. Here we identify SNPs from some of the same genomic regions of cynomolgus macaques (from Indochina, Indonesia, Mauritius and the Philippines) and compare them to the SNPs found in rhesus.

**Results:**

We sequenced a portion of 10 genes in 20 cynomolgus macaques. We identified 69 SNPs in these regions, compared with 71 SNPs found in the same genomic regions of 20 Indian and Chinese rhesus macaques. Thirty six (52%) of the *M. fascicularis *SNPs were overlapping in both species. The majority (70%) of the SNPs found in both Chinese and Indian rhesus macaque populations were also present in *M. fascicularis*. Of the SNPs previously found in a single rhesus population, 38% (Indian) and 44% (Chinese) were also identified in cynomolgus macaques. In an alternative approach, we genotyped 100 cynomolgus DNAs using a rhesus macaque SNP array representing 53 genes and found that 51% (29/57) of the rhesus SNPs were present in *M. fascicularis*. Comparisons of SNP profiles from cynomolgus macaques imported from breeding centers in China (where *M. fascicularis *are not native) showed they were similar to those from Indochina.

**Conclusion:**

This study demonstrates a surprisingly high conservation of SNPs between *M. fascicularis *and *M. mulatta*, suggesting that the relationship of these two species is closer than that suggested by morphological and mitochondrial DNA analysis alone. These findings indicate that SNP discovery efforts in either species will generate useful resources for both macaque species. Identification of SNPs that are unique to regional populations of cynomolgus macaques indicates that location-specific SNPs could be used to distinguish monkeys of uncertain origin. As an example, cynomolgus macaques obtained from 2 different breeding centers in China were shown to have Indochinese ancestry.

## Background

The cynomolgus macaque (*M. fascicularis*) is used widely in biomedical research, advancing the study of simian immunodeficiency virus (SIV) pathogenesis [1-3], transplantation biology [4,5], diabetes [6], and alcohol research [7], among others. Currently, more cynomolgus macaques are imported for use in biomedical research in the United States than are any other non-human primate species. While recent efforts have established many genetic tools for the study of the rhesus macaque, including the complete genome sequence [8], a microsatellite mapping set [9], and a collection of SNPs [10,11], very few genetic resources are available for cynomolgus macaque genetic research [12]. Specifically, the *M. fascicularis *genome sequence, gene expression arrays and a SNP map are not yet available to advance complex trait analysis in cynomolgus macaques.

**Figure 1:**
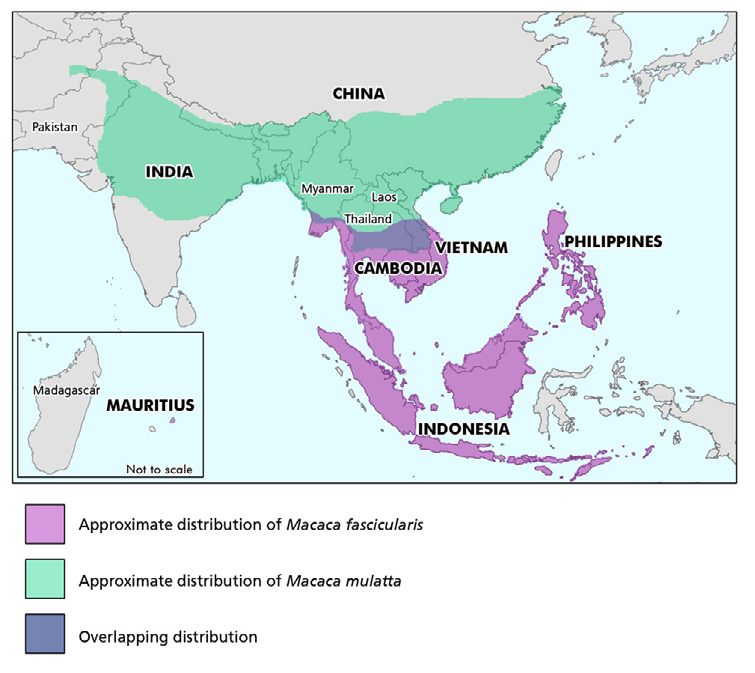
**Geographic ranges of rhesus and cynomolgus macaques**. Geographic locations of rhesus (*M. mulatta*) and cynomolgus (*M. fascicularis*) macaques included in this study are shown in capital letters. Colored shading indicates the approximate distributions for the two species (adapted from Fooden, 1980; and see Fooden, 2000).

Based upon morphological and zoogeographic studies, the cynomolgus macaque and the rhesus macaque are posited to be descendants from a common ancestor inhabiting Indonesia approximately 1.8 million years ago (MYA) [13]. The cynomolgus macaque range currently extends from Indonesia northward through the Philippines and Mainland Southeast Asia, including Myanmar, Thailand and the Indochinese region of Cambodia, Vietnam, Laos (Figure 1) [14,15]. The divergence of the continental and Indonesian animals is estimated to have occurred between 0.4–1.2 million years ago, though periodic hypothermals over the last 250,000 years created land bridges that could have permitted migration between regions [16,17]. An isolated population of cynomolgus macaques inhabits the island of Mauritius. This population is widely considered to have descended from a small number of animals that arrived by trading ships 400–500 years ago [18,19]. This conclusion is supported by analysis of the major histocompatibility complex (MHC) allele distribution in the Mauritian animals, which carry a very limited number of alleles and haplotypes relative to other macaque populations [3,20,21]. Mitochondrial DNA (mtDNA) analysis has shown the Mauritian monkeys to be similar to those of Indonesia and the Philippines [22], and together with Y chromosome sequences, suggest they are specifically derived from Sumatra [23]. Finally, *M. fascicularis *obtained from captive breeding centers in China are commonly used in research throughout the world. However, cynomolgus macaques are not native to China, and thus are descendants of animals imported from elsewhere.

The current range of the rhesus macaque extends from Eastern China through Western India and Pakistan. Within this territory, Indian and Chinese rhesus sub-populations have diverged into genetically distinct subpopulations, as assessed by both mtDNA analysis [24] and MHC allele distributions [25-27]. Recent genomic studies have shown that the vast majority (69%) of SNPs identified in Indian and Chinese rhesus macaque are private to one of the two subpopulations, and that the Chinese population has nearly twice as many SNPs as the Indian rhesus population [10,11]. The dramatic difference in genetic heterogeneity between the Chinese and Indian rhesus macaques has been attributed to both a large expansion of the Chinese population and a contraction of the Indian rhesus population [11].

*M. fascicularis *and *M. mulatta *co-exist in a limited geographic range in Mainland SE Asia. The precise evolutionary history of the macaques in the overlapping range is uncertain. For example, mtDNA sequence comparisons of both species cluster as discrete haplogroups [22,28], consistent with complete lineage sorting and a lack of interspecies hybridization. However the limited genomic sequence comparisons to date, including portions of 4 autosomal loci and 2 Y chromosomal loci, suggest a closer relationship of the species than that predicted by mtDNA analysis [29-31], and suggests possible contemporary gene-flow between the macaques in Indochina [31]. In addition, analysis of the MHC class II region has revealed a high level of allele sharing between rhesus and cynomolgus macaques, though no evidence of identical MHC class II haplotypes [26].

We recently reported the discovery and analysis of Chinese and Indian rhesus macaques SNPs in portions of 94 genes [10]. Here we identified the composition and distribution of SNPs from cynomolgus macaques in 10 of those same gene regions. In addition, a SNP array of rhesus polymorphisms in 53 genes was used to genotype cynomolgus macaque DNAs from 5 different geographic regions (Indochina, Indonesia, Philippines, Mauritius and breeding centers in China). We found 51% of the polymorphisms were present in both cynomolgus and rhesus macaques. These findings suggests that the SNPs identified through genomic discovery efforts in rhesus macaque will have direct benefit to the development of cynomolgus macaque SNP research tools as well.

## Results

**Figure 2:**
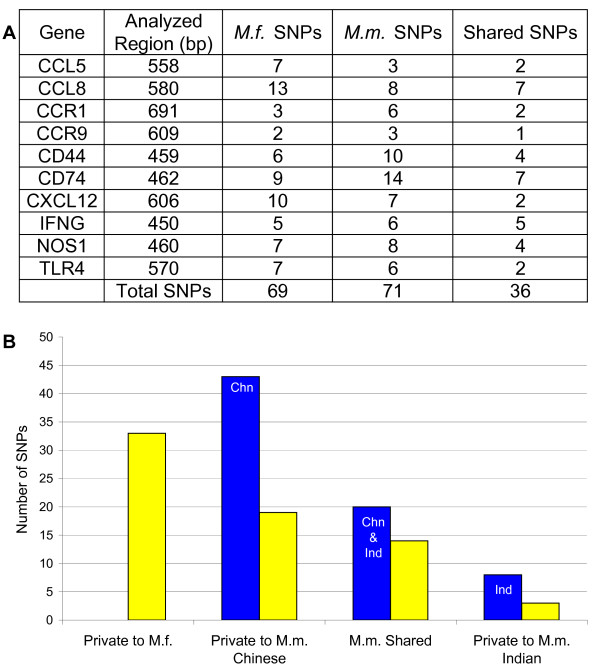
**Comparisons of *M. fascicularis *and *M. mulatta *SNP densities and distributions in 10 genomic regions**. a) The genes in which 3' regions were sequenced in 20 *M. fascicularis *and 20 *M. mulatta *are shown. The number of SNPs found in each species and the number found common to both species are indicated. b) The distribution of SNPs in *M. fascicularis *compared with those found in the same gene regions of *M. mulatta*. The SNPs identified in *M. mulatta *(blue) are grouped into those private to just one rhesus population and those shared by both Chinese and Indian rhesus. The yellow bars indicate the number of SNPs found in *M. fascicularis*.

We sequenced the DNA of 20 cynomolgus macaques, 5 each from Indochina, Indonesia, Mauritius and the Philippines, and compared the sequences to those from the same chromosomal regions of 10 Indian and 10 Chinese rhesus macaques. In each case, the 3' end of a gene was amplified, producing an average DNA fragment of 545 base pairs. The gene regions selected include a representative range of rhesus SNP densities (3–14 SNPs/region) and population distributions of Chinese and Indian rhesus SNPs (63:28). Comparing the *M. fascicularis *and *M. mulatta *sequences, we did not identify any fixed base differences that distinguished the macaque species. The only variants detected were polymorphic. There were a total of 69 SNPs in the cynomolgus macaque DNAs, with individual genes having between 2 and 13 polymorphisms (Figure 2a) (see Additional file 1). This SNP density was almost identical to that of the rhesus macaque, which had 71 SNPs in the same regions.

**Figure 3:**
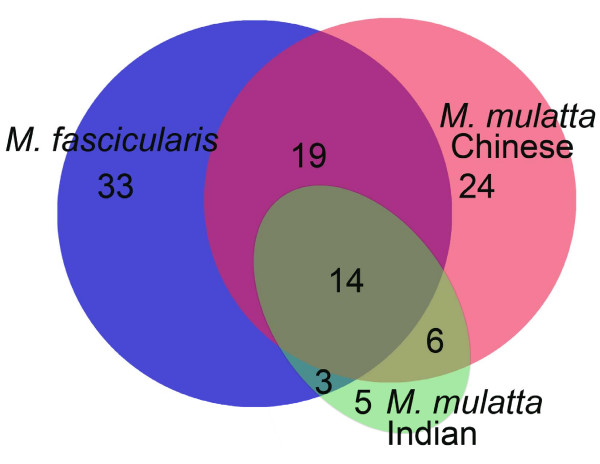
**Venn diagram of SNPs identified in 10 gene regions**. The number and relationship of SNPs identified in *M. fascicularis *and *M. mulatta *through DNA sequencing of 10 gene regions. The numbers of SNPs found in both single and combinations of populations are shown.

A comparison of the SNPs identified in these 10 gene regions revealed a high level of overlap between rhesus and cynomolgus macaques. In total, 52% (36/69) of the SNPs in *M. fascicularis *were also found in rhesus macaques (Figure 2a). All loci sequenced included at least one shared macaque SNP, with an average of 3.6 (SEM ± 0.57) SNPs per locus overlapping between species. Seventy percent (14/20) of the SNPs present in both Indian and Chinese rhesus populations were also present in cynomolgus macaques (Figure 2b). Comparing the two rhesus subpopulations, 38% of the SNPs identified private to Indian rhesus and 44% of those private to Chinese were also found in cynomolgus DNAs. Indian rhesus has an overall lower level of genetic heterogeneity, and thus has a lower absolute number of SNPs that overlap with *M. fascicularis *(17), as compared with Chinese rhesus (33) (Figure 3).

The DNA sequence analysis revealed the level of genetic variation to be similar in all four *M. fascicularis *geographic populations: Indonesia (39), Mauritius (31), the Philippines (29), and Indochina (31) (Table 1) (see Additional file 2). Of these, 36 of the alleles were private to a single geographical population; Indochinese animals had the most private SNPs, 15. To determine if animals from the overlapping range of the two macaque species had a larger number of shared SNPs, we compared the variants in the Indochinese *M. fascicularis *with those from Indonesian animals. We found nearly the same number of SNPs associated with the Chinese rhesus macaques in both Indochinese and Indonesian populations, 19 and 20, respectively (Table 1) (see Additional file 2).

**Table 1:** Distribution of SNPs identified by DNA sequencing 10 gene regions.

	**Total # SNPs**	**Private to *M. f*.**	**Shared with *M. m*. Chinese^2^**	**Shared with *M. m*. Indian^2^**
*M. fascicularis*	69	33	33	17
Indonesia	39 (11)^1^	17	20	11
Mauritius	31 (3)^1^	14	16	9
Philippines	29 (7)^1^	13	15	6
Indochina	31 (15)^1^	11	19	11

^1^The number of SNPs private to each population is noted in parentheses ().

^2^Includes all SNPs identified in this population.

**Figure 4:**
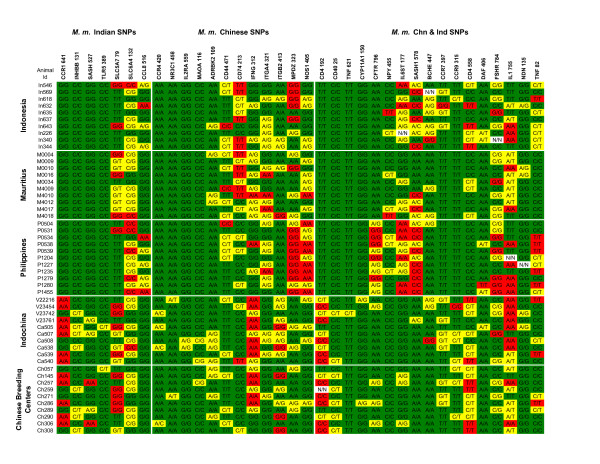
**Genotype analysis of 50 *M. fascicularis *DNAs using *M. mulatta *SNPs**. The *M. fascicularis *populations surveyed are shown on the left. The SNPs are identified at the top, and are grouped by the *M. mulatta *population in which they were originally identified. SNP genotypes identified in *M. fascicularis *are indicated as homozygous major (green), homozygous minor (red), heterozygous (yellow), and no information is indicated by white.

To expand the analysis, DNAs from 100 cynomolgus macaques originating from Indochina (25), Indonesia (20), Mauritius (23), the Philippines (16) and breeding centers in China (16) were genotyped using a SNP array containing previously identified rhesus macaque polymorphisms within the 3'ends of genes [10]. The rhesus SNP array includes 64 SNPs from 53 genes on 18 chromosomes (see Additional file 3). The array incorporates SNPs that have a minimum minor allele frequency (MAF) of 0.15 in at least one rhesus population, and are either unique to Indian rhesus (24), Chinese rhesus (19) or are shared in both Indian and Chinese rhesus populations (21). The genotypes of a representative set of 50 *M. fascicularis *are shown in Figure 4. This assay included 6 SNPs that had been identified in cynomolgus macaques by direct sequencing, and each genotype was confirmed by the SNP array. Excluding the 6 confirmed SNPs, 50% (29/58) of the rhesus SNPs assayed were also present in cynomolgus macaques. In this sampling, 81% of the SNPs previously identified in both Indian and Chinese rhesus were also found in *M. fascicularis*, whereas 63% of the Chinese-specific SNPs and 29% of the Indian-specific SNPs were detected in cynomolgus macaques (Table 2).

**Table 2:** Number of SNPs identified in *M. fascicularis *using an *M. mulatta *SNP array.

	***M. m*. Indian SNPs (24 total)**	***M. m*. Chinese SNPs (19 total)**	***M. m*. Chn & Ind SNPs (21 total)**
Indonesia	3	8	12
Mauritius	2	8	9
Philippines	3	6	9
Indochina	6	12	14
Chinese breeding centers	6	10	12
Total SNPs in *M. fascicularis*	7 (29%)	12 (63%)	17 (81%)

The genotypes of two SNP loci, MAOA:116 and CCR1:641, are of particular interest. MAOA:116 alleles are largely fixed in the rhesus population, with the C-allele being found in Indian rhesus and the G-allele being found in Chinese rhesus [10]. In the cynomolgus macaques 195/200 chromosomes carried the Indian rhesus C-allele. The rare G-allele was only observed in the Indochinese cynomolgus monkeys. At the CCR1:641 locus, the minor allele frequency was high (0.41) in the Indochinese and Chinese *M. fascicularis*, however no heterozygous genotypes were detected among the 41 macaques screened from these populations. The genotypes from both the SNP assay and direct sequencing were concordant at this locus.

We also used the SNP array to compare the genotypes of cynomolgus macaques from known geographic ancestry to those of animals imported from two breeding centers in China. Of the 12 SNPs in this assay that were only detected among the 25 Indochinese macaques, 11 were also found in the 16 animals obtained from China. The average minor allele frequency of these 12 specific SNPs was 0.19 in the Indochinese animals and 0.16 in the *M. fascicularis *from China.

## Discussion

We found that approximately half of the SNPs identified in *M. fascicularis *are also present in *M. mulatta*. The finding indicates that efforts to identify SNPs in either species will be beneficial in generating resources for both macaque species. The recent publication of the rhesus genome sequence has sparked interest in developing genome-wide SNP arrays for use in biomedical research [8]. Such a rhesus macaque SNP array could be used to genotype *M. fascicularis *DNAs, which based upon this analysis, would capture about 50% of the genomic SNPs in cynomolgus macaques.

In this sampling, we identified 36 SNPs that appeared unique to a single geographic population of *M. fascicularis*, suggesting that genetic differences between the populations could underlie unrecognized phenotypic differences between these animals. Since genetic heterogeneity can complicate the reproducibility of results in biomedical studies, it would be prudent to use animals from only a single population of *M. fascicularis *in a single research study. Towards this goal, population-specific SNPs could be used to verify the ancestry of a research animal when it is uncertain. As example, the population-specific SNPs from this study identified cynomolgus macaques from two breeding centers in China as being of Indochinese descent. An expanded SNP discovery effort would readily identify more population markers, making it possible to identify hybrid *M. fascicularis *as well.

The finding that approximately half of the SNPs found in *M. fascicularis *overlap with those found in *M. mulatta *is remarkable, given that previous analysis showed that only 31% of SNPs in rhesus macaques are shared by both the Indian and Chinese subpopulations [10,11]. The overlapping SNPs in *M. fascicularis *include ones that are private to Chinese or Indian rhesus, as well as those that are shared between Indian and Chinese populations. While there are more SNPs from the Chinese rhesus than Indian rhesus macaques present in cynomolgus macaques, there are about twice as many SNPs present in the Chinese rhesus population as whole. An evolutionary bottleneck that reduced the overall genetic diversity of the Indian rhesus macaque has been proposed [11], and such a contraction could also have reduced the representation of ancestral macaque SNPs in Indian rhesus monkeys.

There are several possible explanations for the high percentage of shared variants in these two macaque species. The SNPs identified in both *M. fascicularis *and *M. mulatta *could represent that the most ancient SNPs in the *fascicularis *group, those that predate the divergence of rhesus and cynomolgus macaques. Consistent with this idea, the majority of SNPs found in both Indian and Chinese rhesus macaques were also found in *M. fascicularis*. Interestingly, although mtDNA analysis supports the divergence of these two species 1.8 MYA [32,33], the few studies to date of nuclear DNA loci have suggested a closer relationship [29-31]. Our findings also suggest a more complex evolutionary history than that suggested by mtDNA alone.

It is possible that relatively recent gene flow between these two macaque species has contributed to the high rate of overlap between *M. mulatta *and *M. fascicularis *SNPs. The Y-chromosome sequence studies of Tosi *et al*. suggest interspecies hybridization, though only within the current overlapping range of Chinese rhesus macaques and Indochinese cynomolgus macaques [30,31]. However, in this study we found that Indonesian cynomolgus macaques also share a high percentage of SNPs with the rhesus macaques. Thus if interspecies hybridization contributed to the shared sequences, it likely would have occurred before or during periods of glaciation, when land bridges could have permitted the migration of macaques as far South as Indonesia. Nonetheless, gene flow between Chinese rhesus and *M. fascicularis *does not explain all of the overlapping SNPs, since there is also evidence of Indian-specific rhesus variants in *M. fascicularis*. Due to the geographic barriers that separate India and Indonesia, SNPs common to both of these populations are more likely a consequence of either sequence conservation or convergent evolution.

Selective pressure to maintain some of the sequence variants could have contributed to the retention of some SNPs in both *M. fascicularis *and *M. mulatta*. Possible evidence of selective pressure can be found within this study. By both direct sequence comparisons and SNP array genotyping, we found that one SNP locus (CCR1:641) had a high minor allele frequency (0.41) in the macaques derived from Indochina and Chinese breeding center, and yet no heterozygous individuals were detected, a striking departure from Hardy-Weinberg equilibrium. This finding could be the consequence of inadequate sample size, or a technical issue that was resolved neither by direct sequencing nor by the SNP array. However it is also possible that a heterozygous genotype at CCR1:641, or at alleles tightly linked to this locus, is associated with decreased survival. This is not implausible, since *CCR1 *encodes the chemokine receptor 1 protein, which is involved in leukocyte recruitment in response to pathogenic infections [34].

The MAOA:116 locus has fixed alleles in the Indian and Chinese rhesus macaque populations and was included in this study of *M. fascicularis*. The allele present in > 99% of Indian rhesus (C) was also found in almost all of the *M. fascicularis*, with the only exceptions being a few individuals from the Indochinese population. This skewed presence of the Indian rhesus allele in the *M. fascicularis *animals is striking. The *MAOA *gene encodes monoamine oxidase A, a protein that is involved in the breakdown of neurotransmitters, including norepinephrine and serotonin. Some alleles of *MAOA *are thought to influence aggressive and impulsive behaviors in primates [35]. Perhaps selective pressure favors different *MAOA *alleles in varying macaque populations or environments.

There were no fixed alleles detected in this study that distinguish *M. fascicularis *and *M. mulatta*, and thus there is no direct evidence of gene replacement between species. Based upon the morphological and anatomical differences between the macaques, one would expect some gene replacement to be present. Additional sequencing of larger regions of genomic DNA will be needed to resolve the rate of allele fixation between these two macaque species.

## Conclusion

We found that 52% of the SNPs identified in *M. fascicularis *are also found in *M. mulatta*. The high rate of overlap suggests that the evolutionary relationship of rhesus and cynomolgus macaques may be closer than that suggested by previous morphological and mtDNA analysis. It also indicates that future SNP discovery efforts in either macaque species will generate information that will be useful for both species. Future efforts to identify cynomolgus SNPs would not only advance genetic research in this widely used animal model, but would also generate tools for verifying the origins of animals for studies where ancestry is important.

## Methods

### DNA sources

*M. fascicularis *DNAs used in this study were obtained from at least 2 sources for each geographic region: 25 from Indochina (21 from Cambodia and 4 from Vietnam; SNBL USA, Everett, WA; Alpha Genesis, Yemassee, SC); 20 from Indonesia (Tinjil Island, founded by animals native to Western Java and Southern Sumatra; Java via SNBL USA, Everett, WA; Worldwide Primates, Miami, Florida); 23 from Mauritius (Charles River Laboratories, Wilmington, MA, via the University of Washington and the University of Wisconsin); 16 from the Philippines (SICONBREC, Tanay, Rizal Province, Luzon Island; Del Mundo Trading, Mandaluyong, (central) Manila, Luzon island; Jan Vacek, Located in ILOILO, on the Island of Panay.); 16 from China (SNBL USA, Everett, WA; Alpha Genesis, Yemassee, SC).

### DNA analysis

The 10 gene regions chosen for direct DNA sequence analysis were selected from those previously analyzed in rhesus macaques [10]. In each case, PCR amplification was achieved using Taq Polymerase (Fermentas, Inc., Hanover, MD) in accordance with the manufacturers protocol, along with primers designed from the rhesus macaque genomic sequence (see Additional file 4). Amplification products were separated by agarose gel electrophoresis and isolated using Montage Gel Extraction Kit (Millipore, Inc., Bedford, MA). The DNA fragments were sequenced using the PCR amplification primers and Big Dye Chemistry; the products were separated on a Genetic Analyzer 3130 (Applied Biosystems, Inc., Foster City, CA). Sequence electropherograms were visually inspected and compared using Sequencher 4.7 (GeneCodes, Inc., Ann Arbor, MI).

A custom SNP array was used to genotype 64 previously identified rhesus SNPs, using iPLEX reagents and protocols for multiplex PCR, single base primer extension and generation of mass spectra in accordance with the manufacturer's instructions (Sequenom, Inc., San Diego, CA). The multiplex reactions included 28, 17, 12 and 7 primer sets.

All SNPs identified in this work were deposited in dbSNP and monkeySNP databases [36,37].

## Authors' contributions

BF and RCK conceived of this study. SLS preformed all DNA sequence and statistical analyses and contributed to the writing of the manuscript. RCK provided *M. fascicularis *DNAs and perspective on the project design. RG provided a diverse set of cynomolgus DNAs, as well as insightful review of the results. BF was responsible for the project design, the data analysis and the manuscript preparation. All authors read and approved this manuscript.

## Supplementary Material

Additional file 1 **SNP identity and NCBI accession numbers**. This file lists the SNPs identified in this study and the associated gene. The NCBI STS (reference sequence) accession numbers for each are shown. Click here for file

Additional file 2 **SNPs identified through sequencing in 10 genomic regions of *M. fascicularis *and *M. mulatto***. All SNPs identified in 10 genomic regions by sequencing of 20 *M. fascicularis *and 20 *M. mulatta *are shown at the top. Loci highlighted in green showed no variant allele in that population; yellow highlight indicates a SNP was detected, and red indicates that only the minor allele of the SNP variant was detected in the population. Click here for file

Additional file 3 **SNP assay gene summary**. The fifty-three genes represented in the SNP genotype assay are listed, along with chromosome location. Those genes that were also analyzed by direct sequencing are noted. Click here for file

Additional file 4 **Sequence information for *M. fascicularis***. The primer information and reference sequences for the ten gene regions sequenced in *M. fascicularis *are listed. Click here for file
