# Supplementary material for: Single nucleotide polymorphisms (SNPs) are highly conserved in rhesus (Macaca mulatta) and cynomolgus (Macaca fascicularis) macaques
Source: BMC Genomics. 2007 Dec 31;8:480. doi: 10.1186/1471-2164-8-480 (PMC2248198; doi:10.1186/1471-2164-8-480)
Supplement: Additional file 1 — SNP identity and NCBI accession numbers. This file lists the SNPs identified in this study and the associated gene. The NCBI STS (reference sequence) accession numbers for each are shown. [file 1471-2164-8-480-S1.pdf]

Official Symbol	*Gene Name*	Reference Sequence Accession	SNP	Private to M. fascicularis	Present in M. mulatta
CCL5	*chemokine (C-C motif) ligand 5*	BV723923	CCL5:106		x
CCL5	*chemokine (C-C motif) ligand 5*	BV723923	CCL5:266	x	
CCL5	*chemokine (C-C motif) ligand 5*	BV723923	CCL5:31	x	
CCL5	*chemokine (C-C motif) ligand 5*	BV723923	CCL5:486	x	
CCL5	*chemokine (C-C motif) ligand 5*	BV723923	CCL5:510	x	
CCL5	*chemokine (C-C motif) ligand 5*	BV723923	CCL5:72	x	
CCL5	*chemokine (C-C motif) ligand 5*	BV723923	CCL5:73		x
CCL8	*chemokine (C-C motif) ligand 8*	BV723924	CCL8:219		x
CCL8	*chemokine (C-C motif) ligand 8*	BV723924	CCL8:248		x
CCL8	*chemokine (C-C motif) ligand 8*	BV723924	CCL8:269	x	
CCL8	*chemokine (C-C motif) ligand 8*	BV723924	CCL8:313	x	
CCL8	*chemokine (C-C motif) ligand 8*	BV723924	CCL8:384		x
CCL8	*chemokine (C-C motif) ligand 8*	BV723924	CCL8:452		x
CCL8	*chemokine (C-C motif) ligand 8*	BV723924	CCL8:461	x	
CCL8	*chemokine (C-C motif) ligand 8*	BV723924	CCL8:516		x
CCL8	*chemokine (C-C motif) ligand 8*	BV723924	CCL8:578	x	
CCL8	*chemokine (C-C motif) ligand 8*	BV723924	CCL8:586		x
CCL8	*chemokine (C-C motif) ligand 8*	BV723924	CCL8:611	x	
CCL8	*chemokine (C-C motif) ligand 8*	BV723924	CCL8:618		x
CCL8	*chemokine (C-C motif) ligand 8*	BV723924	CCL8:87	x	
CCR1	*chemokine (C-C motif) receptor 1*	BV723925	CCR1:349	x	
CCR1	*chemokine (C-C motif) receptor 1*	BV723925	CCR1:463		x
CCR1	*chemokine (C-C motif) receptor 1*	BV723925	CCR1:641		x
CCR9	*chemokine (C-C motif) receptor 9*	BV723921	CCR9:315		x
CCR9	*chemokine (C-C motif) receptor 9*	BV723921	CCR9:525	x	
CD44	*CD44 antigen*	BV723926	CD44:234	x	
CD44	*CD44 antigen*	BV723926	CD44:240		x
CD44	*CD44 antigen*	BV723926	CD44:245		x
CD44	*CD44 antigen*	BV723926	CD44:302		x
CD44	*CD44 antigen*	BV723926	CD44:471		x
CD44	*CD44 antigen*	BV723926	CD44:478	x	
CD74	*CD74 antigen*	BV723922	CD74:115	x	
CD74	*CD74 antigen*	BV723922	CD74:124		x
CD74	*CD74 antigen*	BV723922	CD74:138		x
CD74	*CD74 antigen*	BV723922	CD74:186		x
CD74	*CD74 antigen*	BV723922	CD74:213		x
CD74	*CD74 antigen*	BV723922	CD74:247		x
CD74	*CD74 antigen*	BV723922	CD74:270		x
CD74	*CD74 antigen*	BV723922	CD74:339	x	
CD74	*CD74 antigen*	BV723922	CD74:438		x
CXCL12	*chemokine (C-X-C motif) ligand 12*	BV723927	CXCL12:108	x	
CXCL12	*chemokine (C-X-C motif) ligand 12*	BV723927	CXCL12:183	x	
CXCL12	*chemokine (C-X-C motif) ligand 12*	BV723927	CXCL12:234	x	
CXCL12	*chemokine (C-X-C motif) ligand 12*	BV723927	CXCL12:239	x	
CXCL12	*chemokine (C-X-C motif) ligand 12*	BV723927	CXCL12:338	x	
CXCL12	*chemokine (C-X-C motif) ligand 12*	BV723927	CXCL12:492	x	
CXCL12	*chemokine (C-X-C motif) ligand 12*	BV723927	CXCL12:546		x
CXCL12	*chemokine (C-X-C motif) ligand 12*	BV723927	CXCL12:58	x	
CXCL12	*chemokine (C-X-C motif) ligand 12*	BV723927	CXCL12:70	x	
CXCL12	*chemokine (C-X-C motif) ligand 12*	BV723927	CXCL12:95		x
IFNG	*interferon, gamma*	BV723928	IFNG:193		x
IFNG	*interferon, gamma*	BV723928	IFNG:312		x

Official Symbol	*Gene Name*	Reference Sequence Accession	SNP	Private to M. fascicularis	Present in M. mulatta
IFNG	*interferon, gamma*	BV723928	IFNG:391		x
IFNG	*interferon, gamma*	BV723928	IFNG:437		x
IFNG	*interferon, gamma*	BV723928	IFNG:469		x
NOS1	*nitric oxide synthase 1*	BV723929	NOS1:213	x	
NOS1	*nitric oxide synthase 1*	BV723929	NOS1:245		x
NOS1	*nitric oxide synthase 1*	BV723929	NOS1:295		x
NOS1	*nitric oxide synthase 1*	BV723929	NOS1:329		x
NOS1	*nitric oxide synthase 1*	BV723929	NOS1:405		x
NOS1	*nitric oxide synthase 1*	BV723929	NOS1:434	x	
NOS1	*nitric oxide synthase 1*	BV723929	NOS1:437	x	
TLR4	*toll-like receptor 4*	BV723930	TLR4:231		x
TLR4	*toll-like receptor 4*	BV723930	TLR4:320	x	
TLR4	*toll-like receptor 4*	BV723930	TLR4:346	x	
TLR4	*toll-like receptor 4*	BV723930	TLR4:359		x
TLR4	*toll-like receptor 4*	BV723930	TLR4:513	x	
TLR4	*toll-like receptor 4*	BV723930	TLR4:520	x	
TLR4	*toll-like receptor 4*	BV723930	TLR4:641	x	
