# Supplementary material for: Single nucleotide polymorphisms (SNPs) are highly conserved in rhesus (Macaca mulatta) and cynomolgus (Macaca fascicularis) macaques
Source: BMC Genomics. 2007 Dec 31;8:480. doi: 10.1186/1471-2164-8-480 (PMC2248198; doi:10.1186/1471-2164-8-480)
Supplement: Additional file 3 — SNP assay gene summary. The fifty-three genes represented in the SNP genotype assay are listed, along with chromosome location. Those genes that were also analyzed by direct sequencing are noted. [file 1471-2164-8-480-S3.pdf]

Genes analyzed with SNP Assay	Analyzed through direct sequencing	Chromosome
ADRBK2		10
AGRP		20
BCHE		2
CCL5	X	16
CCL8	X	16
CCR1	X	2
CCR4		2
CCR7		16
CCR9	X	2
CCRL1		2
CD4		11
CD40		10
CD40LG		X
CD44	X	14
CD69		11
CD74	X	6
CFTR		3
CX3CR1		2
CXCL12	X	9
CYP11A1		7
DAF		1
FAS		9
FSHR		13
HTATSF1		X
IFNB1		15
IFNG	X	11
IL1		12
IL16		7
IL2		5
IL2RA		9
IL6ST		6
INHBB		12
ITGA4		12
ITGAX		20
ITGB2		3
LRP8		1

Genes analyzed with SNP Assay	Analyzed through direct sequencing	Chromosome
MAOA		X
MPDZ		15
NDN		7
NOS1	X	11
NPY		3
NR3C1		6
PYY		16
SASH1		4
SIRT1		9
SLC5A7		12
SLC6A4		16
SNCA		5
STAR		8
TLR4	X	15
TLR5		1
TNF		4
XCL1		1
