# Supplementary material for: Single nucleotide polymorphisms (SNPs) are highly conserved in rhesus (Macaca mulatta) and cynomolgus (Macaca fascicularis) macaques
Source: BMC Genomics. 2007 Dec 31;8:480. doi: 10.1186/1471-2164-8-480 (PMC2248198; doi:10.1186/1471-2164-8-480)
Supplement: Additional file 4 — Sequence information for M. fascicularis. The primer information and reference sequences for the ten gene regions sequenced in M. fascicularis are listed. [file 1471-2164-8-480-S4.pdf]

Official Symbol	Analyzed Region (bp)	**Forward Primer** (5' to 3')	**Backward Primer** (5' to 3')	Reference Sequence Accession
CCL5	558	GAGGCTTCCCCTCACTATCC	CCAGGAGGAAATCAAACCAA	BV723923
CCL8	580	GGAGAGATGGGTCAGGGATT	CATGGCCAAAAGACATTCCT	BV723924
CCR1	691	AATCCAAAGCTGTCCGTCTG	GTGGGAAATGGGTGACTTTG	BV723925
CCR9	609	GGAGGGAGAAAAGGCTTCTG	CATCAGTGGGGAAACTGCTT	BV723921
CD44	459	GGGAGCTGGGACACTTAACA	TCAAAGGACCTCCAGGGTTT	BV723926
CD74	462	GTCACCCTTGGAAACGAAAA	GGGCTGCATTCTCTCACCTA	BV723922
CXCL12	606	CAGAGGGGCTGAATAGCAGA	CCATGTCCAGAAAATTCCAA	BV723927
IFNG	450	TCCTGTGACTGTTTCACTTAATCC	CATTTAATGGAACCAAATGCAA	BV723928
NOS1	460	CTCAGGGTGGGAAGAACTTG	TGTTGGTGCCCTCATTGTAA	BV723929
TLR4	570	CAAGATGCCCCTTCAATTTT	CTGGCAGTGAAGAGGGTACA	BV723930
