# Supplementary material for: Single nucleotide polymorphisms (SNPs) are highly conserved in rhesus (Macaca mulatta) and cynomolgus (Macaca fascicularis) macaques
Source: BMC Genomics. 2007 Dec 31;8:480. doi: 10.1186/1471-2164-8-480 (PMC2248198; doi:10.1186/1471-2164-8-480)
Supplement: Additional file 2 — SNPs identified through sequencing in 10 genomic regions of M. fascicularis and M. mulatto. All SNPs identified in 10 genomic regions by sequencing of 20 M. fascicularis and 20 M. mulatta are shown at the top. Loci highlighted in green showed no variant allele in that population; yellow highlight indicates a SNP was detected, and red indicates that only the minor allele of the SNP variant was detected in the population. [file 1471-2164-8-480-S2.pdf]

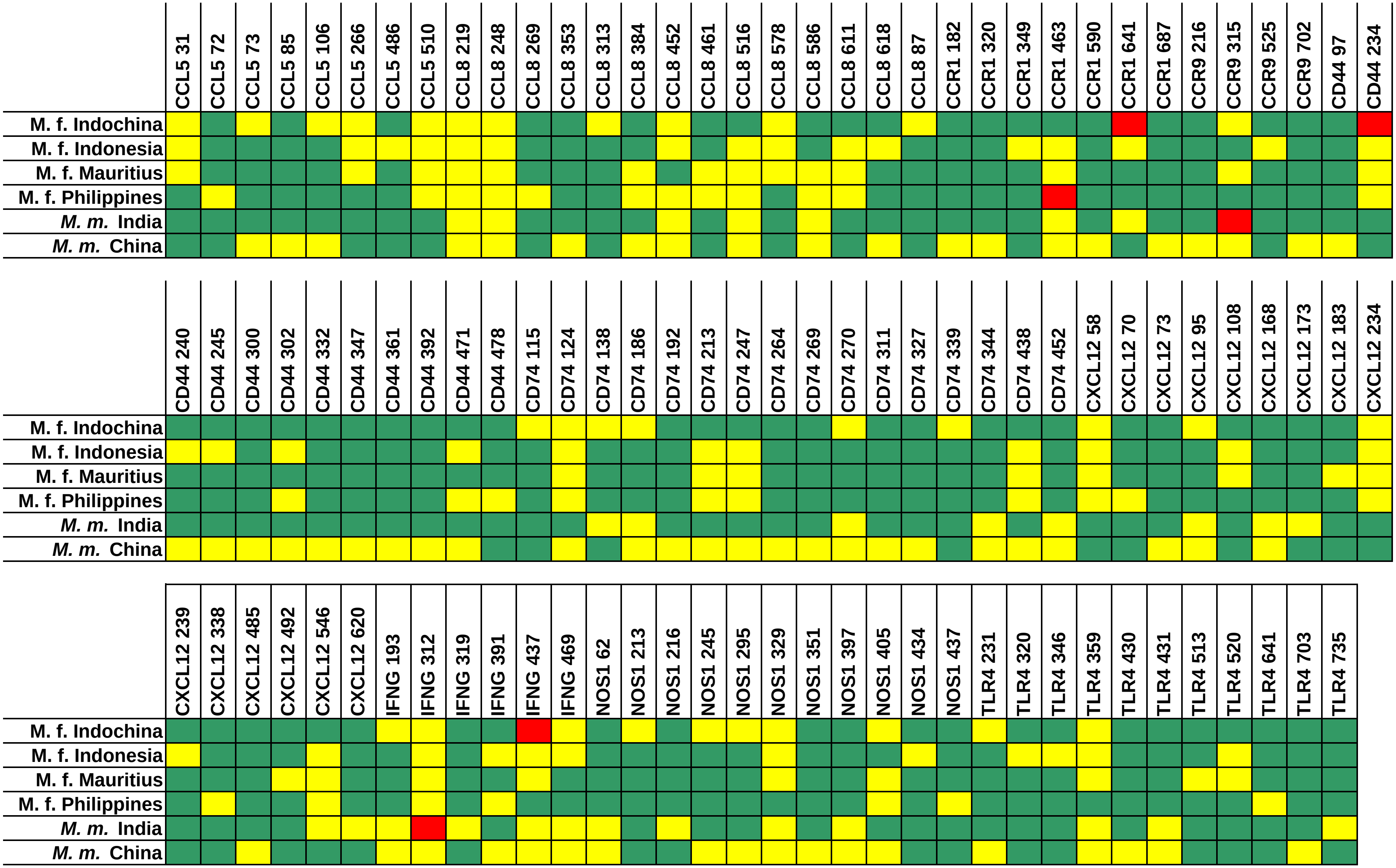
CCL5 31
CCL5 72
CCL5 73
CCL5 85
CCL5 106
CCL5 266
CCL5 486
CCL5 510
CCL8 219
CCL8 248
CCL8 269
CCL8 353
CCL8 313
CCL8 384
CCL8 452
CCL8 461
CCL8 516
CCL8 578
CCL8 586
CCL8 611
CCL8 618
CCL8 87
CCR1 182
CCR1 320
CCR1 349
CCR1 463
CCR1 590
CCR1 641
CCR1 687
CCR9 216
CCR9 315
CCR9 525
CCR9 702
CD44 97
CD44 234
M. f. Indochina
M. f. Indonesia
M. f. Mauritius
M. f. Philippines
M. m. India
M. m. China
CD44 240
CD44 245
CD44 300
CD44 302
CD44 332
CD44 347
CD44 361
CD44 392
CD44 471
CD44 478
CD74 115
CD74 124
CD74 138
CD74 186
CD74 192
CD74 213
CD74 247
CD74 264
CD74 269
CD74 270
CD74 311
CD74 327
CD74 339
CD74 344
CD74 438
CD74 452
CXCL12 58
CXCL12 70
CXCL12 73
CXCL12 95
CXCL12 108
CXCL12 168
CXCL12 173
CXCL12 183
CXCL12 234
M. f. Indochina
M. f. Indonesia
M. f. Mauritius
M. f. Philippines
M. m. India
M. m. China
CXCL12 239
CXCL12 338
CXCL12 485
CXCL12 492
CXCL12 546
CXCL12 620
IFNG 193
IFNG 312
IFNG 319
IFNG 391
IFNG 437
IFNG 469
NOS1 62
NOS1 213
NOS1 216
NOS1 245
NOS1 295
NOS1 329
NOS1 351
NOS1 397
NOS1 405
NOS1 434
NOS1 437
TLR4 231
TLR4 320
TLR4 346
TLR4 359
TLR4 430
TLR4 431
TLR4 513
TLR4 520
TLR4 641
TLR4 703
TLR4 735
M. f. Indochina
M. f. Indonesia
M. f. Mauritius
M. f. Philippines
M. m. India
M. m. China
